# Cold Baths in Typhoid Fever

**Published:** 1891-04-18

**Authors:** 


### The Practitioner's Mirror and Retrospect.

[*The Editor will be glad to receive offers of co-operation and contributions from members of the profession. All letters should be addressed to* The Editor, The Lodge, Porchester Square, London, W.]

### MEDICINE.

### COLD BATHS IN TYPHOID FEVER.

Of all the specific fevers none present greater difficulties in arriving at the amount of benefit attributable to any particular method of treatment than enteric fever, for in none does the severity of symptoms in different epidemics vary so greatly as in this affection. It is only by a very long series of observations and a careful numerical analysis of the results we can come to a conclusion of any real value. The question of cold baths in the treatment of typhoid fever is an example of these difficulties, for in spite of the special attention this question has received, opinion as to the value of such treatment is much divided. The systematic treatment of typhoid fever by cold baths was advocated nearly a century ago by Currie, but fell into disuse till the method was revived by Brand, of Stettin, and since taken up by many physicians in Germany and in this country. Dr. Cayley recorded his results in his Croonian Lectures about ten years ago.

The grounds on which the advocates of antipyretic treatment of fevers take their stand is the injurious results that prolonged high temperature produces in the tissues, and not the fact that high temperature, by increasing metabolism, increases the tissue waste. As a matter of fact, immersion in cold water actually increases the amount of heat evolved—at all events, for a time. But there are certain changes which prolonged high temperature induces in the tissues, and a return to the normal cannot counterbalance these evil effects entirely. The fact that heart myosin coagulates at about a temperature of 105°, or thereabouts, has been urged in favour of the antipyretic treatment; but however heart myosin may behave when extracted from the tissues, it is very certain that living heart myosin in the body does not coagulate at this temperature.

Dr. Barr's recent series of lectures on the treatment of this disease by prolonged immersion in water draws attention to the value of this new modification of the older methods of antipyretic treatment. The patients are immersed in a tank, which consists of a well-made wooden box, six feet long by two feet ten inches wide, and a foot in depth. It is lined with lead. Each tank is provided with a large discharge pipe, which, in the case of these tanks, communicates with a soil pipe, which leads down to the sewer; thus the tank, containing seventy gallons of water, can be emptied in three minutes. Each tank is provided with a sheet of bed ticking, which would about allow the patient to be submerged, but at the head there is a strip, about a foot wide, which does not sink so deeply, and on which rests an air pillow, so as to keep the head above water. The patient is wrapped up in a blanket and completely immersed except the head. It is important, Dr. Barr says, to use a blanket for this purpose rather than a sheet, because frequently a small portion of the chest rises above the water, and the blanket being a conductor of heat, this portion of the surface does not get chilled, which would happen if a sheet were used. The tank is covered with half a lid, so as to prevent the weight of the clothes resting on the patient, and a waterproof sheet and bed clothing to keep in the heat of the water. A thermometer is kept constantly in the tank. As long as the patient's temperature is over 100°, the temperature of the tank need not rise above 90° to 93°; but as the body temperature approaches the normal, so should the tank temperature. He has not found it necessary to lower the temperature of the water below 90°, nor to raise it above 98°. A rise of a few degrees in the tank temperature is sure to send up the heat of the body, though to a less extent, and by regulating the heat of the water there is no fear of any collapse, as the temperature of the body cannot fall below that of the surrounding medium. The duration of the patient's stay in the bath varied from six to thirty-one days, and in every case it was tried a cure resulted. The patients were kept chiefly on a milk diet, and none of them had alcohol in any form during the course of treatment, and very little medicine. No antipyretics were given, and there was no attempt to prevent the usual evening exacerbation, but this was gradually lessened in intensity and duration, and the remissions became greater and longer. The pulse became slower, fuller, and of improved tension, and the respirations lessened in frequency; the bronchitis and congestion of the lungs improved and soon disappeared. The improvement in the digestive tract was perhaps more marked than anywhere else, the tongue became clear and moist, the appetite and digestion improved, and the diarrhœa not only lessened, but the character of the motions changed for the better.

The general tendency of medical practice is to employ cold and other antipyretics—not as a systematic treatment of pyrexia, but to combat hyperpyrexia as it occurs. It is far from clear that a prolonged moderately high temperature, up to about 103°, is in itself a source of much harm, and it is certainly open to doubt whether more harm is not done by attempts to reduce it. It is very possible that a certain amount of pyrexia is beneficial in its action in such diseases as enteric fever, and though the employment of antipyretics in this affection is based on extensive and careful clinical observations, and not, as is so frequently the case in new methods of treatment, on isolated observations in the physiological laboratory, it cannot be too clearly understood that the whole question is still *sub judice.* We must refer, however, to Dr. Hare's article in the *Practitioner*, giving his results in the cold bath treatment of pneumonia, for he has been able to realise those conditions which are requisite in order that the statistics of results may be reliable. His observations have been carried on during the last four years and a-half, at the Brisbane Hospital. Of 586 consecutive cases treated almost entirely by the expectant method [only two or three of these cases bathed], 85 died, giving a percentage mortality of 14 5. Towards the end of the time during which these cases were observed quinine was rather freely used in antipyretic doses. Cold sponging was frequently used, and the cold wet sheet not uncommonly in cases with persistent high temperature. On referring to 1,828 cases treated in that hospital during the previous four years and a-half, 271 had proved fatal, or a percentage of 14·82, and from this Dr. Hare concluded that it might be fairly assumed that practically there was no advance being made in results.

The 586 cases with a mortality of 14·5 per cent. are regarded by Dr. Hare as a "control list whereby to demonstrate the results obtained from the introduction of the bath treatment." From January 1, 1887, down to December 31, 1889, every case admitted to the hospital, in which the rectal temperature reached 102·2° F., was submitted to a systematic course of cold bathing in the absence of the usual contra-indications, viz., perforation, peritonitis, intestinal hæmorrhage; diarrhœa, meteorism and hæmorrhage were treated by the constant application of icebags to the abdomen in addition to the ordinary measures. The results in 1,173 cases treated thus in the course of three years was 92 deaths, *i.e.*, a percentage mortality of 7·84, or an improvement of fifty per cent on the older statistics.

Dr. Hare discusses fully and most fairly the fallacies that may militate against the value of these figures, and shows how these fallacies have been in the present instance sufficiently minimised, if not entirely eliminated, and we may attach great importance to his conclusions, which we therefore quote *in extenso.* "(1) That by means of the bath treatment, systematically employed, the hospital death rate of typhoid may be greatly reduced; (2) that the reduction should amount to 50 per cent. on the previous death rate, and that the percentage mortality to admissions should not be over 8 per cent.; (3) that this result may be obtained in spite of the fact that many of the cases are unsuited to the treatment, and that much more might be expected in appropriate cases only; (4) that the success is in proportion as the treatment is begun early in the disease; (5) that, as evidenced by the undiminished occurrence of perforation and hæmorrhage, and by the fact that early admission has failed to render them less frequent, the treatment has no influence on the depth of the ulceration; (6) that since a constant percentage (about 4¼) of the cases admitted are from these accidents, no reduction in the general mortality much below 5 per cent. can be expected from the treatment, even were it possible to ensure every case being admitted under the most favourable circumstances; (7) that as the result of the different liability of the sexes to these accidents, the prognosis under the bath treatment is vastly more favourable in females than in males; (8) that on the whole the lethal influence of the intestinal lesion is diminished; that the treatment effects this (*a*) directly, by moderating diarrhœa, and (*b*) indirectly, by sustaining the powers of the patient, and thereby enabling him to recover from the effects of the hæmorrhage and other not necessarily fatal intestinal conditions; (9) that the vast bulk of the reduction in mortality is due to the prevention of those complications and modes of death, which being more or less common to the febrile state, however induced, have been termed pyrexial. Thus (*a*) fatal pneumonia has been less than one-fourth as frequent, this being chiefly due to the rarity of the bronchial form; (*b*) brain complications have been less fatal, and brain symptoms (delirium, stupor, &c.) enormously reduced in frequency; while (*c*) it is no exaggeration to say that simple cardiac failure would have been practically expunged from the list had all the cases admitted come under treatment during the first week of the disease."

